# Estimates of country level temperature-related mortality damage functions

**DOI:** 10.1038/s41598-021-99156-5

**Published:** 2021-10-13

**Authors:** R. Daniel Bressler, Frances C. Moore, Kevin Rennert, David Anthoff

**Affiliations:** 1 School of International and Public Affairs, Columbia University, New York City, USA; 2 The Earth Institute at Columbia University, New York City, USA; 3 Center for Environmental Economics and Policy, Columbia University, New York City, USA; 4 Department of Environmental Science and Policy, University of California Davis, Davis, USA; 5 Resources for the Future, Washington, DC USA; 6 Energy and Resources Group, University of California at Berkeley, Berkeley, USA

**Keywords:** Climate-change impacts, Climate-change impacts, Environmental economics, Risk factors

## Abstract

Many studies project that climate change is expected to cause a significant number of excess deaths. Yet, in integrated assessment models that determine the social cost of carbon (SCC), human mortality impacts do not reflect the latest scientific understanding. We address this issue by estimating country-level mortality damage functions for temperature-related mortality with global spatial coverage. We rely on projections from the most comprehensive published study in the epidemiology literature of future temperature impacts on mortality (Gasparrini et al. in Lancet Planet Health 1:e360–e367, 2017), which estimated changes in heat- and cold-related mortality for 23 countries over the twenty-first century. We model variation in these mortality projections as a function of baseline climate, future temperature change, and income variables and then project future changes in mortality for every country. We find significant spatial heterogeneity in projected mortality impacts, with hotter and poorer places more adversely affected than colder and richer places. In the absence of income-based adaptation, the global mortality rate in 2080–2099 is expected to increase by 1.8% [95% CI 0.8–2.8%] under a lower-emissions RCP 4.5 scenario and by 6.2% [95% CI 2.5–10.0%] in the very high-emissions RCP 8.5 scenario relative to 2001–2020. When the reduced sensitivity to heat associated with rising incomes, such as greater ability to invest in air conditioning, is accounted for, the expected end-of-century increase in the global mortality rate is 1.1% [95% CI 0.4–1.9%] in RCP 4.5 and 4.2% [95% CI 1.8–6.7%] in RCP 8.5. In addition, we compare recent estimates of climate-change induced excess mortality from diarrheal disease, malaria and dengue fever in 2030 and 2050 with current estimates used in SCC calculations and show these are likely underestimated in current SCC estimates, but are also small compared to more direct temperature effects.

## Introduction

The social cost of carbon (SCC) is arguably the single most important concept in the economics of climate change^1^. It quantifies the net cost of emitting one additional metric ton of carbon-dioxide-equivalent at a certain point in time^2^. According to standard economic theory, it represents the price that should be put on carbon dioxide to reduce emissions to socially optimal levels along the optimal emissions trajectory^3^. The SCC is used in several countries to inform the cost–benefit analysis of climate and energy policy. Regulations with benefits totaling over $1 trillion in the United States have used the SCC in their economic analysis^1^.

Despite the theoretical and policy importance of the SCC, the representation of current scientific understanding of climate damages in the integrated assessment models (IAMs) used to calculate it is lacking^4,5^. In particular, climate change impacts on human health and mortality are both a critical aspect of climate change costs and not well represented in current SCC estimates. For example, the Climate Framework for Uncertainty, Negotiation and Decision (FUND) model accounts for mortality through a number of different pathways, but its estimates are based off of studies from the 1990s and 2000s^6^ while the scientific evidence base for mortality impacts in the Dynamic Integrated Climate and Economy (DICE) model is also dated^7^. A 2017 National Academy of Sciences (NAS) report specifically mentioned mortality as an impact sector that could be readily updated in IAMs^8^.

Improving estimates of the mortality costs of climate change is particularly timely given a large body of scholarly literature that has improved understanding of the relationship between future temperature change and excess mortality. For instance, studies have assessed the effect of climate change effects on mortality from heat-related mortality^9–15^, changes in air pollution^16,17^, cold-related mortality^9,13,14^, and disease burdens^12,18^. A Lancet report concluded that “Climate change is the biggest global health threat of the twenty-first century”^19^ while a recent study estimated that anthropogenic warming over the 1991–2018 period is responsible for 37% of warm-season heat-related deaths^20^. Incorporating this literature into SCC estimates can have important implications for estimated damages: A recent study found that revising global mortality estimates increased the SCC by a factor of 7 in the single-region DICE model^7^.

A fuller accounting of mortality impacts in the SCC however, requires damages at higher spatial resolution, since several IAMs used to calculate the SCC model multiple regions^6,21^. This study fills this gap in the literature by extrapolating projections of heat- and cold-related mortality from the most comprehensive published study in the epidemiology literature to date—a 2017 Lancet Planetary Health Study by Gasparrini et al^9^—to produce country-level mortality damage functions. We estimate the mortality damage function both with and without accounting for the projected benefits of higher incomes on reducing vulnerability to heat. Our approach is fully consistent with recommendations by the NAS for calculating the SCC, which recommended that damage functions should be based on the current, peer-reviewed literature on climate impacts, have uncertainties that are characterized and quantified where possible, and be transparent, well-documented, and reproducible^8^. The NAS also recommended that damages should be given in natural/physical units which, for mortality costs, is the number of excess deaths associated with carbon dioxide emissions^7^. Finally, we also discuss climate change effects on mortality other than direct temperature effects. We compare estimates of climate-change impacts on diarrheal disease, malaria and dengue from a 2014 World Health Organization (WHO) report by Hales et al.^12^ with those currently incorporated into SCC estimates.

## Methods

Our approach relies on extrapolating from previous projections for climate change effects on heat- and cold-related mortality for 23 countries published in Gasparrini et al. 2017^9^ to the global scale. Therefore, the following section provides a more detailed description of the methods and findings from that study. Subsequent sections describe the extrapolation approach used in this paper.

### Gasparrini et al. 2017: methods and results

Our temperature-related mortality damages rely on results reported in Table S2 of Gasparrini et al^9^. The study estimates country-specific statistical relationships between daily temperature and mortality for 23 countries. These countries are selected based on availability of daily mortality data and together make up around 40% of the world’s population. High income countries such as those in Europe are well-represented, but the study also makes projections for some low to middle income and hotter countries, including Thailand, the Philippines, Brazil, China, Mexico, and Vietnam. (A full list of countries included in the Gasparrini et al. (1) study is: Canada, USA, Mexico, Brazil, Chile, Finland, Ireland, Sweden, UK, Czech Republic, France, Moldova, Switzerland, Italy, Spain, China, Japan, South Korea, Philippines, Taiwan, Thailand, Vietnam and Australia.) Dates of data availability vary between countries, but extend between 4–5 years (i.e. Vietnam and the Philippines) and 24 years (i.e. the United States), over the period 1984–2015.

The authors model the relationship between daily mortality and mean daily temperatures using a 21-day nonlinear distributed lag model, chosen to capture both the known lagged effects of exposure to cold on mortality and the harvesting effect^22^. They estimate the country-specific effect of daily temperature on mortality, allowing for different responses to hot and cold temperatures.

They then combine these response functions with projections of changes in the daily temperature distribution over the twenty-first century to estimate country-specific changes in mortality for the periods 2050–2059 and 2090–2099 under four different emissions scenarios (i.e. Representative Concentration Pathways or RCPs). For all 23 countries, Gasparrini et al. predict an increase in heat-related excess mortality and a decrease in cold-related excess mortality under climate change scenarios, with most countries experiencing a net increase in mortality. It is these 368 projections (23 countries, hot and cold-related mortality, two time points, and 4 RCPs) that are the basis of our extrapolation to other countries.

### This study

Because CO_2_ is a global pollutant, it is essential that economic accounting of climate change damages, including SCC estimates, include costs to all countries. Therefore, while Gasparrini et al. provide valuable empirical evidence regarding the effects of temperature change on mortality rates for the 23 countries in that study, incorporating this evidence into the SCC requires extrapolating their findings to the global scale. Here we provide that global extrapolation, by modeling the estimated change in heat- and cold-related mortality given in the original study as a function of countries’ climatology and income, using these relationships to project climate change effects on mortality for all countries.

#### Data

We use population-weighted historical climate data at the country-level using the University of Delaware dataset and the 2015 population given by the Gridded Population of the World Dataset v4^23,24^. For climate projection data, we use country-level projections provided by the World Bank Climate Knowledge portal produced by an ensemble of CMIP5 models^25^. For historical and projected per-capita income data, we use shared socioeconomic pathways (SSP) projections available from the International Institute for Applied Systems Analysis^26^.

#### Model selection

Because Gasparrini et al. report heat- and cold-related mortality separately, and because it is plausible that both the physiological mechanisms (i.e. pathways by which heat and cold affect human health) and socio-economic factors determining vulnerability (i.e. ability to protect oneself from extreme temperature through home heating or air conditioning for example) differ between the two, we treat these two sources of mortality impacts separately. We consider the following set of variables that might plausibly explain variance in the mortality impacts projected by Gasparini et al.: amount of warming (both linear and non-linear effects); interaction of the amount of warming with the hottest or coldest month of the year in that country (since warming would be expected to have larger effects in places with more extreme temperatures); and interaction with per-capita income, to capture the mediating effect of income on vulnerability to extreme temperatures. For both types of impacts, we test the performance of several models that account for non-linearities in the effect of warming and interactions with country climate and income. We examine both in and out of sample performance but focus on out of sample performance because the goal is extrapolation to other countries.

### Heat-related mortality

We estimate the percentage increase in the mortality rate due to heat-related mortality as a function of the increase in yearly average temperatures at the country level. In the Gasparrini et al. paper, this projection is available for 23 countries for both mid-century and end of century for four RCPs (resulting in 184 data points). We test four possible models explaining the variation in projected heat-related mortality across countries and warming-levels. Model 1 is a simple linear function of projected warming. Model 2 allows for a non-linear effect of warming. This allows for the exponential increase in extreme hot days (i.e. exceedance of some threshold) expected in a given place as a function of that area’s average temperatures^27,28^. Model 3 allows for an interaction with the average temperature in the hottest month of the year from 1984 to 2015. This allows the same amount of warming to have a different effect in places that are already hot relative to cooler locations^22^. Finally, Model 4 adds an additional interaction term with per-capita income, reflecting the ability of individuals and groups to make investments that mitigate the negative mortality effect of heat, such as installing air conditioning^13,29^. Standard errors are clustered at the country level, allowing for residual correlation between observations for the same country.

We tested multiple model specifications containing these variables, shown in Table 1. We found that Model 4 performed the best on both in-sample measures and Leave One Out Cross-Validation (LOOCV), achieving a root mean square error of 2.38 and a mean absolute error of 1.29 in LOOCV. Model 4 takes into account the projected temperature increase, the current climate, and current income levels, and it is represented by the following equation:

1 $$\begin{aligned} Y\_Hot_{s,c,t} & = \beta_{1} T_{s,c,t} + \beta_{2} T_{s,c,t}^{2} + \beta_{3} T_{s,c,t} *Hottest\,Month\,Avg\,Temp_{c} \\ & \quad + \beta_{4} T_{s,c,t} *Hottest\,Month\,Avg\,Temp_{c} *\log \left( {GDPPC_{c} } \right) + \varepsilon_{s,c,t} \\ \end{aligned}$$

**Table 1:** Heat model validation.

N	(1)	(2)	(3)	(4)
	184	184	184	184
**In-sample validation**				
adj. R-sq	0.458	0.456	0.543	0.600
F	28.72	13.69	14.89	11.23
Rmse	2.621	2.625	2.407	2.251
**Leave one out cross-validation**				
Rmse	2.645	2.666	2.480	2.382
Mean absolute errors	1.466	1.455	1.308	1.287
Psuedo-R-sq	0.202	0.181	0.292	0.348
**Covariates**				
Projected_Temperature_Increase	1.940*** (0.400)	2.463*** (0.508)	− 1.943 (1.673)	− 0.532 (1.224)
Projected_Temperature_Increase_2		− 0.200 (0.0989)	− 0.145 (0.0771)	− 0.0629 (0.0935)
Projected_Temperature_Increase*HottestMonthAvgTemps			0.196* (0.0839)	0.525* (0.208)
Projected_Temperature_Increase*HottestMonthAvgTemps*log_GDP_Per_Capita_PPP				− 0.0409 (0.0222)

Heat model specification (4) performed the best out of the models both on in-sample validation and out of sample validation.

Standard errors in parentheses *p < 0.05, **p < 0.01, ***p < 0.001.

The subscript $c$ represents the country. The subscript $s$ represents the scenario, which represents whether the projection is for RCP 2.6, 4.5, 6.0, or 8.5. The subscript *t* represents whether the projection is for mid-century or end of century. When there is no scenario or time subscript, this implies that the variable is an observed variable for the present period. $Y\_Hot_{s,c,t}$ is the percentage increase in the mortality rate due to heat estimated by Gasparrini et al. $T_{s,c,t}$ is the increase in yearly average temperatures relative to the present period (specifically the 2001–2020 average) for country $c$ in scenario $s$ in time $t$. $HottestMonthAvgTemp_{c}$ is the current population-weighted average temperature in the hottest month in a given country and $\log \left( {GDPPC_{c} } \right)$ is the current per-capita income in country c. Note the absence of a constant term, which forces the response through the origin, required for interpretation of the estimated effect as a climate damage function (i.e. no climate change implies no climate change damages^30^.

Table 1 shows evidence for a worsening effect of higher levels of warming, based on the positive $\beta_{2}$ coefficient on $T_{s,c,t}^{2}$. There is also evidence that hotter countries are expected to on average be more harmed for a given temperature increase than currently colder countries, as is shown by the positive $\beta_{3}$ coefficient on the interaction with hottest monthly temperature. We also see a negative interaction with $\log \left( {GDPPC_{c} } \right)$ (i.e. a negative $\beta_{4}$ coefficient) indicating that richer countries can ameliorate some of the damages associated with higher temperatures. This could well be associated with air conditioning penetration, which several studies have shown to be strongly associated with higher incomes, particularly in warmer, middle-income countries^31,32^.

This income interaction is important in projecting future mortality effects of climate change, as per-capita incomes are projected to grow over time. If wealthier populations are better able to protect themselves from extreme heat, not accounting for this expected income growth risks over-estimating climate change damages. We use Model 4 to estimate mortality effects both with and without accounting for the expected beneficial effects of rising incomes (based on SSP3). The former allows a comparison to Gasparrini et al. who did not model the effects of rising incomes, while the latter allows for this potential income-based adaptation pathway.

### Cold-related mortality

We performed a similar exercise to estimate the percentage decrease in the mortality rate due to cold-related mortality as a function of the increase in yearly average temperatures at the country level. Gasparrini et al. also made this projection for 23 countries for both mid-century and end of century for four RCPs (resulting in 184 data points). As shown in Table 2, we ran the same model specifications as for heat-related mortality, except that we now use the population-weighted average temperatures in the coldest month instead of the hottest month in the interaction terms.

**Table 2:** Cold model validation.

N	(1)	(2)	(3)	(4)
	184	184	184	184
**In-sample validation**				
adj. R-sq	0.856	0.868	0.911	0.911
F	137.8	95.57	119.7	89.87
Rmse	0.574	0.548	0.450	0.451
**Leave one out cross-validation**				
Rmse	0.578	0.557	0.461	0.465
Mean absolute errors	0.427	0.424	0.355	0.357
Psuedo-R-sq	0.446	0.450	0.625	0.617
Projected_Temperature_Increase	− 0.977*** (0.0902)	− 1.569*** (0.131)	− 1.441*** (0.137)	− 1.441*** (0.137)
Projected_Temperature_Increase_2		0.226*** (0.0356)	0.199*** (0.0361)	0.199*** (0.0366)
Projected_Temperature_Increase*Coldest_Month_Avg_Temps			− 0.0113 (0.00680)	− 0.0127 (0.0610)
Projected_Temperature_Increase*Coldest_Month_Avg_Temps*log_GDP_Per_Capita_PPP				0.000146 (0.00626)

Cold model specification (3) performed the best out of the models both on in-sample validation and out of sample validation. Unlike the heat model, adding in the interaction with log GDP per capita did not improve the model performance.

Standard errors in parentheses *p < 0.05, **p < 0.01, ***p < 0.001.

Unlike for heat-related mortality, we found that adding in the interaction term that included log GDP per capita (i.e. Model 4) slightly worsened performance for cold-related mortality both in-sample and in out-of-sample LOOCV. For cold-related mortality, Model 3 thus provided the best fit. Due to less variation in the dependent variable between countries, the cold-related mortality models are more precise than the heat-related mortality models, with Model 3 achieving a root mean square error of 0.47 and a mean absolute error of 0.36. Model 3 is represented by the following equation:

2 $$\begin{array}{*{20}c} {Y\_Cold_{s,c,t} = \beta_{1} T_{s,c,t} + \beta_{2} T_{s,c,t}^{2} + \beta_{3} T_{s,c,t} *Coldest\,Month\,Avg\,Temp_{c} + \varepsilon_{s,c,t} } \\ \end{array}$$

where the terms in the equation are the same as Eq. (1), except now $Y\_Cold_{s,c,t}$ represents the percentage change in the mortality rate due to cold and $ColdestMonthAvgTemp_{c}$ is the yearly average temperature in the coldest month.

After estimating both $Y\_Hot_{s,c,t}$ and $Y\_Cold_{s,c,t}$, $Y\_Net_{s,c,t} = Y\_Hot_{s,c,t} + Y\_Cold_{s,c,t}$ is the percentage change in the mortality rate due to the net effects of changes to heat and cold related mortality. The percentage increase in the mortality rate is relative to a counterfactual scenario in which no additional warming occurs and the contribution of heat-related and cold-related mortality towards the mortality rate remains constant at the present level.

**Figure 1:**
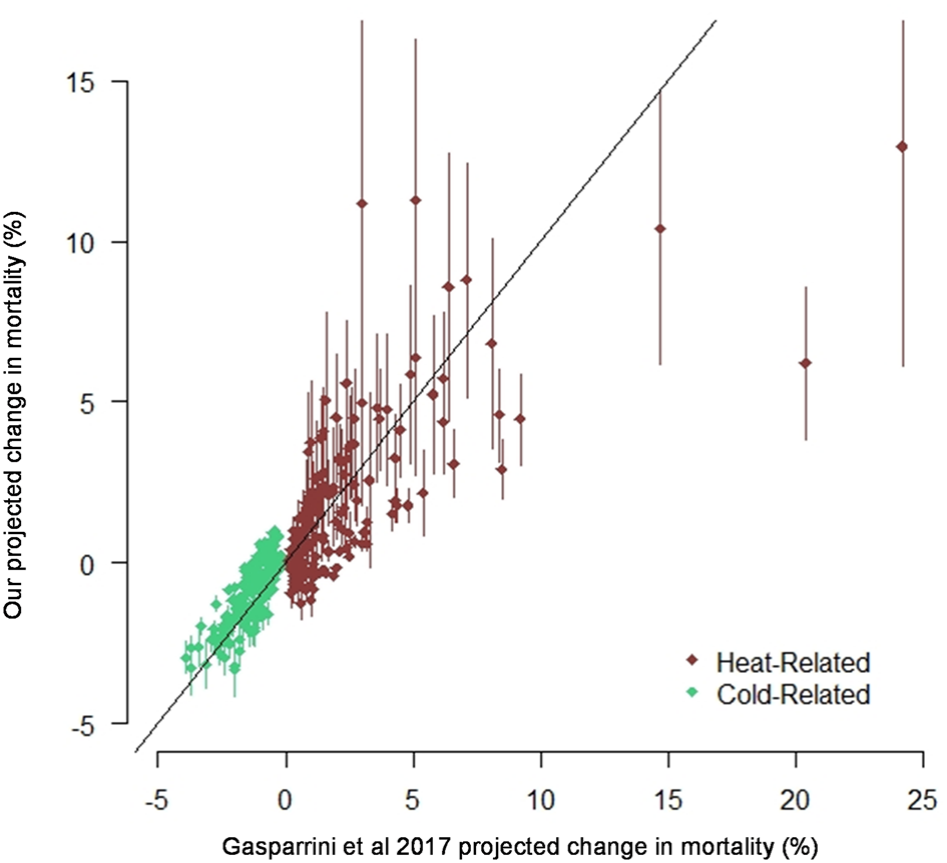
Heat and Cold Model Projections vs. Gasparrini 2017. 95% confidence intervals for our estimates are shown on the vertical lines.

We evaluate the performance of our two preferred models by showing our fitted value for the original 164 projections (23 countries × 4 RCPs × mid and end of century estimates) made by Gasparrini et al. Figure 1 shows estimates of $Y\_Hot_{s,c,t}$ (red) and $Y\_Cold_{s,c,t}$ (green) against the original data. Points falling along the one-to-one line (plotted on Fig. 1) are a perfect match. As discussed, our model achieved a closer fit for cold-related mortality relative to heat-related mortality because there is less variation in cold-related mortality estimates between countries and since the overall magnitude of reductions in cold-related mortality is generally smaller compared to projected increases in heat-related mortality. The chart also shows three heat-related mortality outliers where Gasparrini et al.’s projections are significantly higher than our projections: Thailand in the end of century RCP 8.5 projection (+ 14.7), Mexico in the end of century RCP 8.5 projection (+ 20.4), and Vietnam in the end of century RCP 8.5 projection (+ 24.2). However, it is important to note that error bars reported in Gasparrini et al. (Table S2 of that paper) for these estimates are wide—in all three cases our central estimate is well within the 95% confidence interval of the original projection.

## Results

### Temperature-related mortality

**Figure 2:**
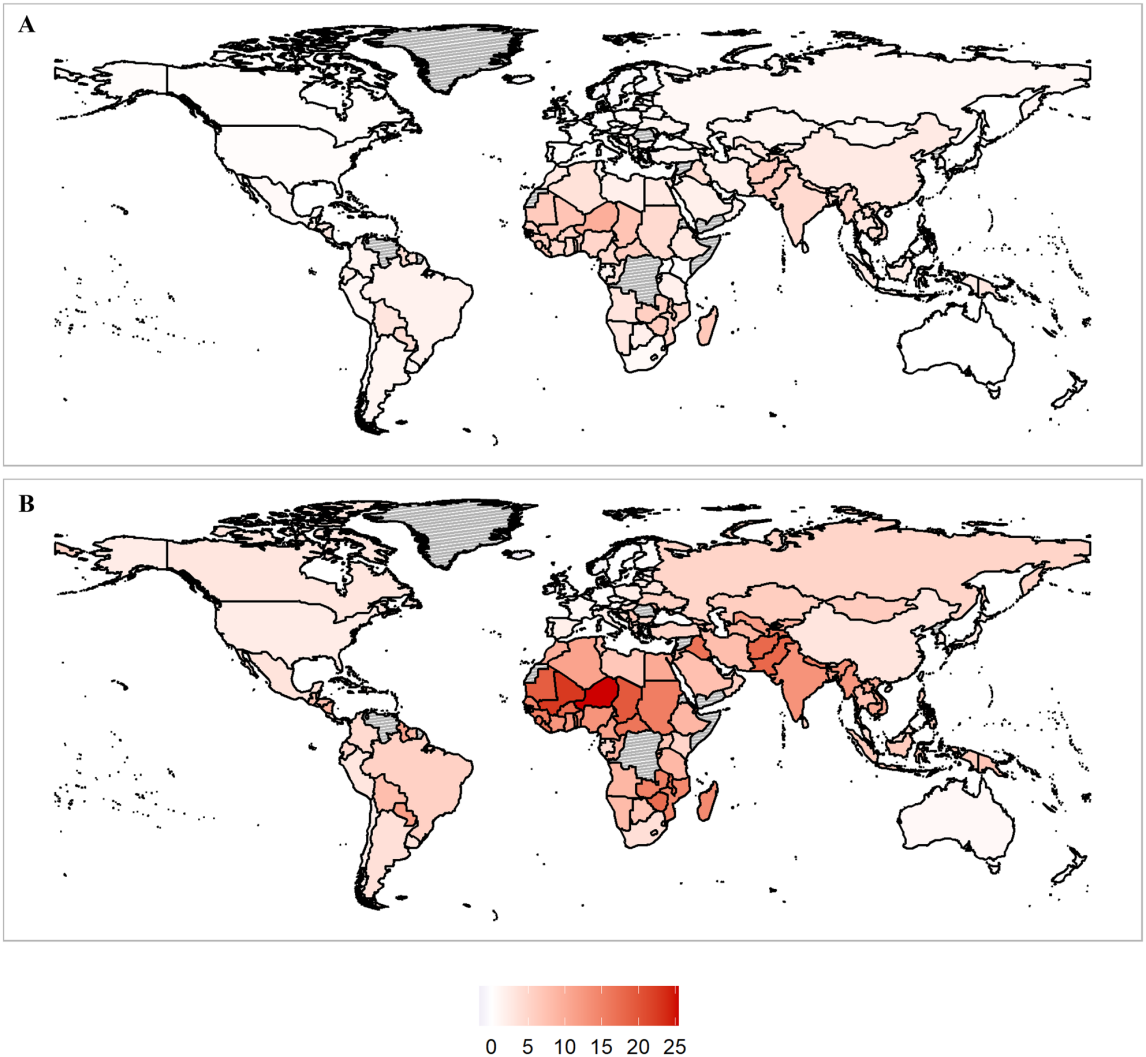
Net increases in the mortality rate due to temperature-related mortality (%). (**A**) RCP 4.5 end of century. (**B**) RCP 8.5 end of century. Gray countries indicate countries where sufficient data wasn't available from the World Bank to make projections. Maps were produced by the authors using R v.3.6.3 (https://www.r-project.org/).

Using the preferred models for heat- and cold-related mortality described in the previous section, we project a mortality damage function for every country based on the expected warming and their observable characteristics (namely, hottest and coldest monthly temperatures and income). The results are shown in Fig. 2, which shows a map of the increase in mortality at the end of the century for RCP 4.5 (panel a, a 2.2 °C increase in global average temperatures above preindustrial) and RCP 8.5 (panel b, a 4.3 °C increase in global average temperatures above preindustrial). As the map shows, the projected impact of climate change on temperature-related mortality is significant under high levels of warming, and highly heterogenous between countries.

The net impact of temperature-related mortality ranges from a − 1.6% [95% CI − 4.3–1.0%] decrease in the mortality rate for Iceland to a 25.5% [6.5–44.5%] increase in the mortality rate for Niger in RCP 8.5 at the end of century. Places that are generally hotter and poorer are expected to have the largest increase in mortality—especially Africa, the Middle East, and Southern Asia. Some countries in Northern Europe are expected to get a small mortality benefit. A CSV file with projections for every country in RCP 2.6, RCP 4.5, RCP 6.0, and RCP 8.5 in the mid-twenty-first century and the end of the twenty-first century is provided as a supplement.

**Figure 3:**
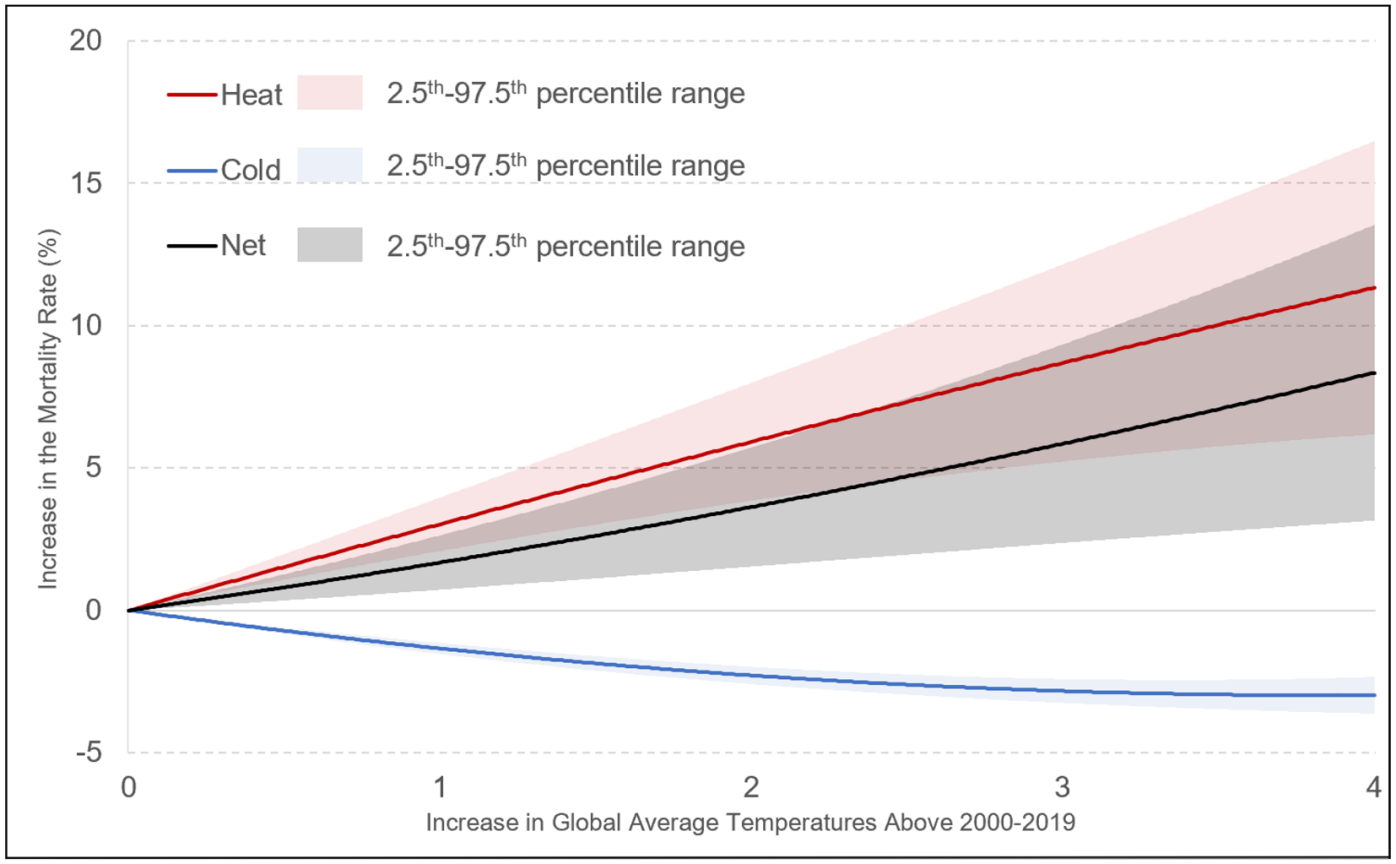
Increases in the global mortality rate as a function of the increase in global average temperature (without income-based adaptation). Under climate change, heat is expected to cause an increase in mortality (red line) while cold is expected to cause a decrease in mortality (blue line). The effect of heat is expected to outweigh the effect of cold, and the net result of these two effects is an increase in the mortality rate (black line). At 4 °C warming above the correct level, the mortality rate is expected to increase by 8.4% relative to a counterfactual scenario in which there is no warming and the contribution of temperature-related mortality towards the mortality rate remains constant at the present level.

Figure 3 shows the increase in global mortality as a function of the increase in global average temperatures. As shown, the increase in the mortality rate from increasing heat exposure is larger than the decrease in the mortality rate from reduced exposure to cold temperatures, and the net effect is an increase in the mortality rate. In addition, there is much greater uncertainty in heat-related mortality projections than the cold-related mortality projections.

#### Temperature-related mortality accounting for income-based adaptation

**Figure 4:**
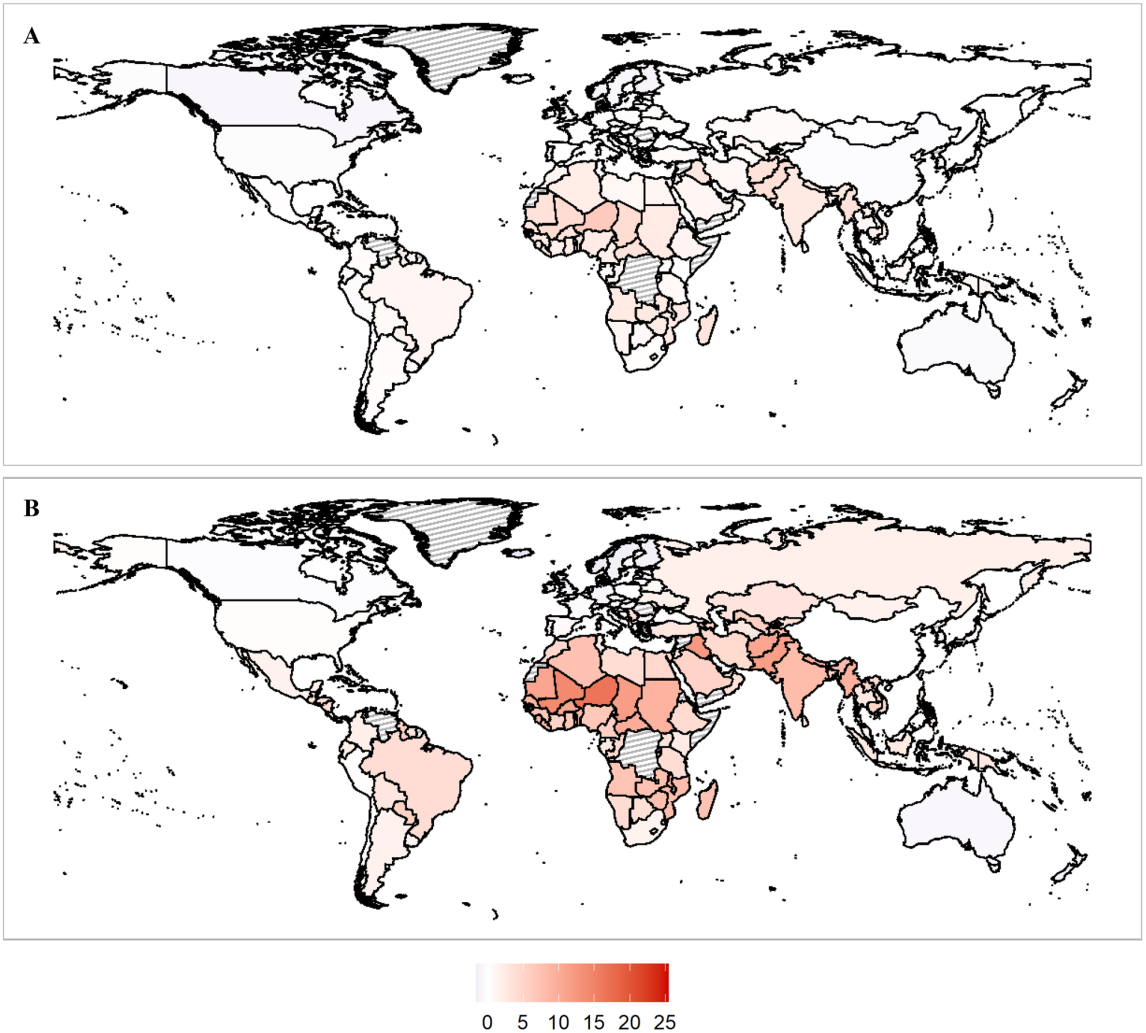
Net increases in the mortality rate due to temperature-related mortality with income-based adaptation (%). (**A**) RCP 4.5 end of century. (**B**) RCP 8.5 end of century. Gray countries indicate countries where sufficient data wasn't available from the World Bank to make projections. Maps were produced by the authors using R v.3.6.3 (https://www.r-project.org/).

We also provide projections of temperature-related mortality that account for income-based adaptation as discussed in the methods section. To project future income, we use SSP3. The results of this exercise are shown in Fig. 4, which shows a map of the increase in mortality at the end of the century for RCP 4.5 (panel a) and RCP 8.5 (panel b). As the figure shows, income-based adaptation is expected to provide some benefit in mitigating temperature-related mortality, but the increase in mortality remains substantial for many countries in higher emissions scenarios. Assuming income-based adaptation results in a projected − 2.1% [− 4.6–0.4%] decrease in the mortality rate in Iceland, a 16.7% [6.2–27.2%] increase in the mortality rate in Niger.

**Figure 5:**
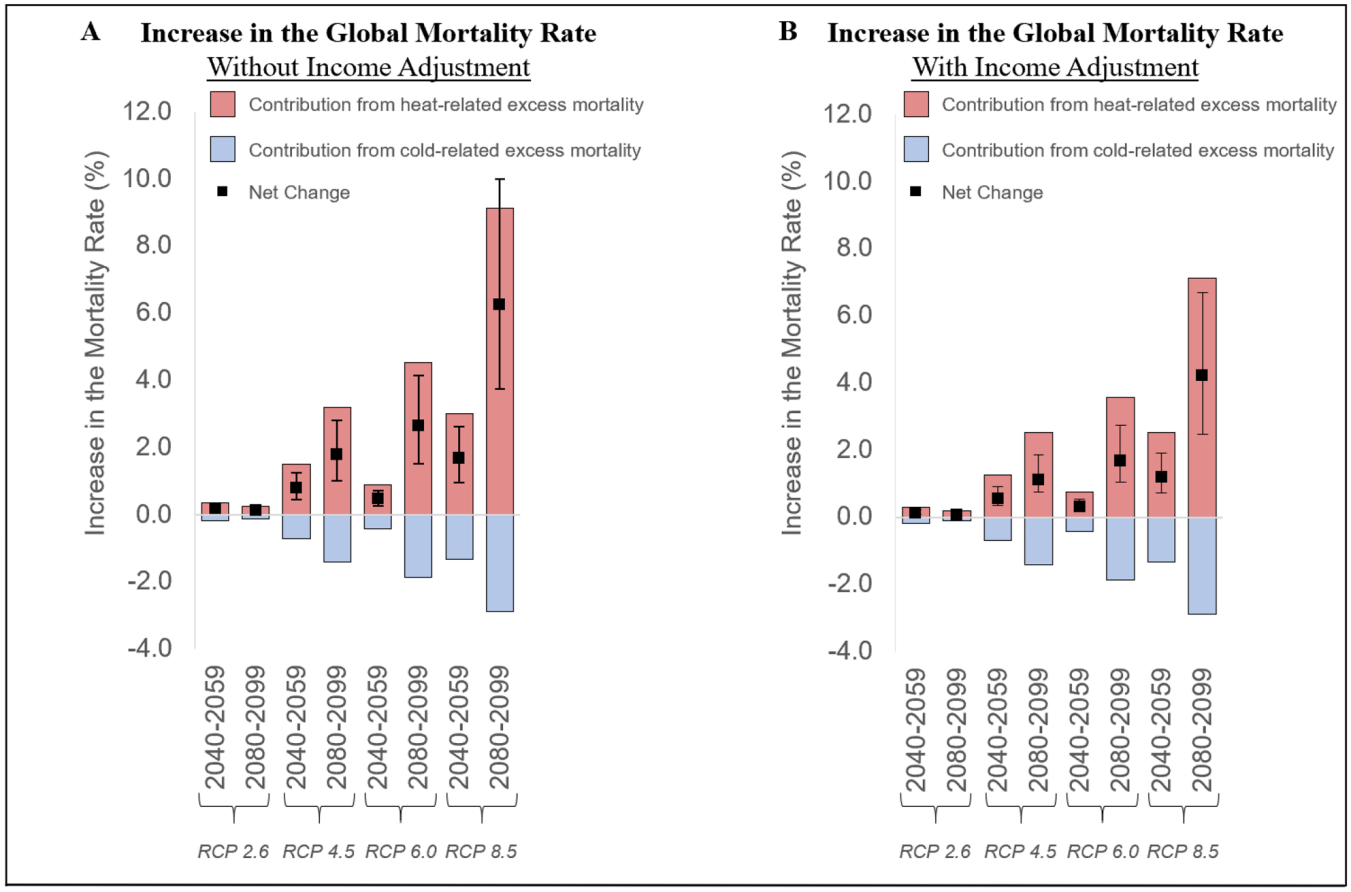
Increase in the global mortality rate by scenario. The graphs show the contribution of global warming towards the mortality rate through its effect on temperature related mortality, without (Panel **A**) and with (Panel **B**) including the estimated protective effects of higher incomes, projected using SSP3. The black error bars show 95% CIs for the net mortality impact.

Figure 5 shows the effect of income-based adaptation on the global projections. Higher incomes play a role in mitigating the effect of climate change, although the global mortality rate increases by at least 1% at the end of the century in all the RCP scenarios except for RCP 2.6. The mortality rate remains especially elevated in the high emissions RCP 8.5 scenario, although income-based adaptation reduces the mortality rate increase from a 6.2% increase in the mortality rate [95% CI 2.5–10.0%] to 4.2% [95% CI 1.8–6.7%].

It is possible that other forms of adaptation and technological changes, other than through income-based adjustments, might modify the temperature-mortality relationship over time. For example, provision of public heat alert systems, improved preparation of the medical system for heat-related diseases, or people learning to avoid activity during the hottest parts of the day might all reduce the adverse effects of extreme heat over time. Several studies reviewed by Arbuthnott et al.^33^ show evidence of decreasing sensitivity of heat-related mortality over time. These effects are not included in the estimates given here.

#### Other climate change-induced impacts on mortality

The impacts of climate change quantified above based on Gasparrini et al. are those associated directly with changes in temperature. Climate change is also expected to affect mortality through a number of other, more indirect pathways. These include deaths attributable to storms or flooding caused by or exacerbated by climate change, deaths from the spread of vector-borne diseases due to higher temperatures, and deaths due to under-nutrition from reduced crop yields. It is not straightforward to account for these more complex pathways relating climate change and mortality in a sector-based, bottom-up accounting of climate change impacts such as that used for SCC estimates. One example of an IAM that attempts to do so is the FUND model^6^, which includes both an agricultural and storms damage component intended to account for the mortality and morbidity costs of changes to extreme events and agricultural yields.

Here we focus on two additional components of climate change-induced mortality—deaths due to changes in vector-borne diseases and changes in diarrheal disease—which are both quantified explicitly in the FUND model and for which more recent projections exist. In a 2014 WHO report Hales et al. report results of several modeling studies that projected excess deaths due to climate change for 21 world regions and five impact pathways (undernutrition, malaria, dengue, diarrhea, and heat stress for over-65 s) in both 2030 and 2050 under an A1B emissions and socio-economic scenario^12^.

Findings reported in Hales et al.^10^ for diarrheal and vector-borne diseases result from interactions between changing population, increasing incomes, and changing temperature and rainfall patterns. Given these interaction effects and the data provided in the report, attempting to infer regional damage functions from the information provided is beyond the scope of this study. However, Table 3 gives the global values (and uncertainty ranges) reported in Hales et al. and compares them to excess deaths for the same impacts projected by FUND for the same time-points under the A1B scenario.

**Table 3:** Global excess mortality (number of deaths) due to climate change effects on diarrheal disease and vector-borne diseases for 2030 and 2050 under the A1B scenario from Hales et al.^12^.

	Diarrheal disease		Vector-borne diseases			
			Malaria		Dengue	
	2030	2050	2030	2050	2030	2050
Central estimate	48,100	33,000	60,100	32,700	260	280
Range	21,100–67,700	14,900–49,200	37,600–117,000	22,800–40,800	140–330	200–340
FUND	33,800	14,500	5400	1000	30	10

Range gives minimum and maximum values. Values from the FUND 3.9^19^ model’s diarrheal disease and vector-borne impact components for the same time-points under A1B are also provided.

Note that in almost all cases the climate change effects on these diseases declines over time, despite larger levels of warming later in the century. This is due to interactions with projected income growth: given historical precedents in wealthier countries, it is highly likely that base-rates of these diseases will fall over time with rising incomes. Hales et al. incorporate this as a projected fall in the rate of communicable disease over time (diarrheal disease) and with a per-capita income term in the models of vector-borne disease prevalence. FUND explicitly includes an income-elasticity term in modeling climate change effects for all three diseases.

Table 3 also provides evidence that FUND is likely under-estimating climate-change impacts within these sectors, particularly for vector-borne diseases and particularly later in the century, compared with estimates in Hales et al. Vector-borne disease impacts in FUND are lower by an order of magnitude in 2030 and that disparity grows in 2050. FUND impacts for diarrheal disease are very close to those in Hales et al. (and well within the uncertainty range) in 2030, but are half the magnitude in 2050. It seems probable that these growing disparities are due to larger income effects in FUND, though a lower sensitivity to temperature change cannot be ruled out.

However, a final point to note is that these effects on mortality are small compared to the direct heat- and cold-related mortality effects described in the previous sections. Hales et al. project a total of 65,000 excess deaths in 2050 due to effects on diarrheal disease, malaria and dengue. In contrast, applying our model to the global mortality rate, RCP 4.5 mid-century warming is projected to increase the global mortality rate by 0.6%, after accounting for the protective effect of rising incomes (Fig. 4b). Given mortality projections under the medium fertility variant of the UN Population Prospects^34^, this corresponds to an additional 580,000 deaths in 2050, nine times larger than those from these other impact pathways.

## Discussion and conclusion

In this study, we created country-level temperature-related mortality damage functions by extrapolating the results of the Gasparrini et al. to every country to estimate the effect of climate change on the mortality rate through its effect on temperature-related mortality. Here, our implied global mortality damage function projects that in RCP 8.5 end of century (2080–2099), there will be a projected 4.2% increase in the global mortality rate due to temperature-related mortality when the protective effects of rising incomes are accounted for. This global average is quantitatively similar to a global mortality damage function reported recently in Bressler^7^ based on a meta-analysis of published studies, which projects 4.4% increase in mortality by RCP 8.5 in 2090 as well as the working paper by Carleton et al.^13^, which reports a 5.1% increase in temperature-related mortality by 2090 under RCP 8.5.

The results of this paper can be used to inform IAM damage functions, by embedding our Eqs. (1) and (2) along with the estimated coefficients directly in IAMs to dynamically project the increase in the mortality rate from climate change in different emissions scenarios and socio-economic projections at the country level. The projected increase in the mortality rate can then be multiplied by the country’s projected mortality rate and population size (which may be determined endogenously by the IAM, or which can be estimated from outside projections, such as the UN Population Prospects) to estimate the number of excess deaths from climate change^34^. We also provide the raw data for our mid and end of twenty-first century mortality projections for nearly every country in the world in RCP 2.6, 4.5, 6.0, and 8.5 as a supplementary data file. Updating IAMs with the results from this study will help to ensure that social cost of carbon estimates are updated to the latest science on temperature-related mortality^8^.

## Supplementary Information

Supplementary Legends.

Supplementary Table S1.
