# Supplementary material for: Estimates of country level temperature-related mortality damage functions
Source: Sci Rep. 2021 Oct 13;11:20282. doi: 10.1038/s41598-021-99156-5 (PMC8514527; doi:10.1038/s41598-021-99156-5)
Supplement: Supplementary file 1 — Supplementary Legends. [file 41598_2021_99156_MOESM1_ESM.docx]

**Table S1:** Projected changes in mortality rate for heat- and cold-related mortality using the preferred statistical models (described in the main text), for 163 countries, for mid- and end-of-century under four different RCPs with standard errors. Projections of heat-related mortality are presented with and without accounting for projected changes in per-capita income over the 21^st^ century (SSP3).
